# Algorithmic approaches to ostomy management: An integrative review

**DOI:** 10.1002/nop2.1044

**Published:** 2021-08-31

**Authors:** Corey Heerschap, Britney Butt

**Affiliations:** ^1^ Royal Victoria Regional Health Centre Barrie ON Canada; ^2^ School of Nursing Queens University Kingston ON Canada; ^3^ North York General Hospital Toronto ON Canada

**Keywords:** algorithm, integrative review, literature review, nursing, ostomy algorithm, ostomy management, ostomy nurse, ostomy pathway, stoma management, stoma nurse

## Abstract

**Objective:**

The aim of this review is to describe approaches to ostomy management utilizing algorithmic approaches found within the literature.

**Design:**

An integrative review approach was used based on a modified Cooper's five‐stage research review framework.

**Data Sources:**

Systematic searches occurred using the CINAHL and MEDLINE databases searching for peer‐reviewed, English publications.

**Review Methods:**

There were 640 articles identified through the review process, 608 of which were excluded based on title and abstract review. The remaining 12 articles were assessed in full text after which two studies were removed as duplicates and six studies were excluded based on inclusion/exclusion criteria. Four studies were included in this synthesis. Studies were critically analysed using a critical appraisal tool developed for both qualitative and quantitative study assessments.

**Results:**

Utilizing inductive content analysis, included literature was presented within two categories: validation of ostomy algorithms and implementation of ostomy algorithms in practice. Four themes emerged from these categories including the following: algorithm validation, identifying underlying causes, focus on accessories and large‐scale implementation.

**Conclusion:**

No currently available validated algorithms published in full were found during this literature review. Current literature demonstrates the potential benefit for ostomy management algorithms to standardize and improve ostomy patient care.

**Impact:**

This study sought to determine the availability and supporting research of ostomy management algorithms which may assist in standardizing and improving ostomy care. This review has demonstrated a lack of available ostomy management algorithms. Given the potential benefit of ostomy algorithms identified within the literature, further studies should be completed to develop, validate and test new ostomy management algorithms.

## 1 INTRODUCTION

There are as many as 1.3 million individuals living with an ostomy worldwide (Registered Nurses Association of Ontario [RNAO], 2019). An ostomy is a surgical opening in the abdomen resulting in the external diversion of faeces or urine (RNAO, 2019). The creation of an ostomy can have profound effects on both the physical and psychological well‐being of the patient along with negatively impacting quality of life (RNAO, 2019). With as many as 71% of individuals with an ostomy experiencing complications, there is a clear need for a standardized approach to assist caregivers in determining appropriate ostomy management (RNAO, 2019). Algorithms, or the graphical display of decision logic and sequence of events, has been discussed as leading to improved understanding of content, increased knowledge retention and can lead to increased use of clinical best practices (Rosenfield et al., 2012). Although pathways and guidelines have been developed outlining management of the ostomy patient prior to, during, and after their ostomy operation, these documents often focus on education and support, or a general checklist of tasks for care. While these pathways are very beneficial to improving practices, they often do not assist clinicians in the management of the patient's ostomy itself (Lovejoy et al., 1997; Miller et al., 2017). When discussion of ostomy management does occur within a guideline, it is not done in an algorithmic fashion (RNAO, 2019). This literature review will focus on algorithmic approaches to ostomy management identified within the literature.

## 2 BACKGROUND

The decision of which pouching system should be used to manage a patient's ostomy is considered one of the greatest difficulties facing the clinician (Turnbull, 2005). A proper ostomy appliance can prevent complications or manage a current issue which has arisen. Ostomy pouching systems, as we know them today, have only been available since after the mid‐1950’s (Turnbull, 2003). Since this time, ostomy pouches and accessories have expanded and evolved significantly with multiple options to consider when managing a patient's ostomy.

The patient's own choice of pouching system will play an important role in determining products used (Turnbull, 2002). That being said, management of the ostomy and choice of the pouching system and accessories should accomplish a number of important tasks. These include protecting the peristomal skin through containment of effluent, controlling odour, allowing for self‐management and providing a manageable and consistent wear time (Turnbull, 2002).

Complications arising from ineffective or inappropriate use of ostomy management equipment can have a profound effect on the patient's quality of life and result in financial strain (LeBlanc, et al., 2019; RNAO, 2019). Examples of ostomy complications include issues such as contact dermatitis, denudement, peristomal wounds, folliculitis and fungal infections (RNAO, 2019). These issues are often managed by correcting leakage through pouching system changes such as convexity or accessory use (RNAO, 2019). Sixty per cent of those who experience ostomy‐related issues identify that they seek out specialized ostomy nurses, who are nurses who have obtained additional education in ostomy management, which has been shown to lead to decreased ostomy supply utilization (LeBlanc, et al., 2019). The availability of these specialists however is limited and often ostomy care is provided by the non‐specialized nurse, who does not have the same ability to determine appropriate management of an ostomy as a specialized nurse (Beitz et al., 2010). It has been recognized that, given the importance of proper ostomy management and the impact it has on the individual with an ostomy, a standardized algorithmic approach to management, easily followed by the non‐specialist nurse, would be of benefit (Beitz et al., 2010).

## 3 AIM

Our aim in this review is to describe approaches to ostomy management utilizing algorithmic approaches found within the literature. The PICO mnemonic was used to create the research question: “What algorithmic approaches are available to assist in the management of ostomies?”. The population in this instance is individuals with an ostomy, and the intervention is the algorithmic approach, while the outcome is the effect these algorithms have had on ostomy management. No comparison was included within this study.

## 4 DESIGN

An integrative literature review method as outlined by Whittemore and Knafl (2005) was used during the design and implementation of this manuscript. This method is a modified version of Cooper's 1998 five‐stage research review framework (Whittemore & Knafl, 2005). The five stages of conducting an integrative review include the following: a problem formulation stage, a literature search stage, a data evaluation stage, a data analysis stage and a presentation stage (Whittemore & Knafl, 2005). The initial stage of problem formulation occurred when seeking out options for ostomy management algorithm integration within the author's own practice. Recognizing limited available options, with varying methods of evaluation, it was felt an integrative review to collect information on available algorithmic approaches would be appropriate.

## 5 LITERATURE SEARCH METHODS

### 5.1 Eligibility criteria

The inclusion criteria for studies within this review were as follows: (a) the study was written in English, (b) the study was peer‐reviewed, and (c) the study contributed to knowledge about ostomy management using an algorithmic approach. The exclusion criteria included the following: (a) secondary literature, (b) position statements, (c) abstracts, (d) editorials or (e) grey literature. During the eligibility assessment of selected articles, all literature was reviewed in full text to determine eligibility against the inclusion and exclusion criteria.

### 5.2 Information sources and literature search

A literature search was conducted using the CINAHL and MEDLINE databases. The database search occurred during the month of December 2020. A search was conducted using the key terms, “Ostomy” AND “Algorithms OR Decision Trees OR Pathway.” MeSH‐terms were used in the MEDLINE search, and CINAHL Headings were used when searching CINAHL when available. The search limiters included the following: “English language” and “peer‐reviewed.” In the MEDLINE search, we were unable to limit by peer review, and thus, these articles had their peer review criterion assessed individually during full‐text review. No time limit was set.

## 6 SEARCH OUTCOME

**FIGURE 1:**
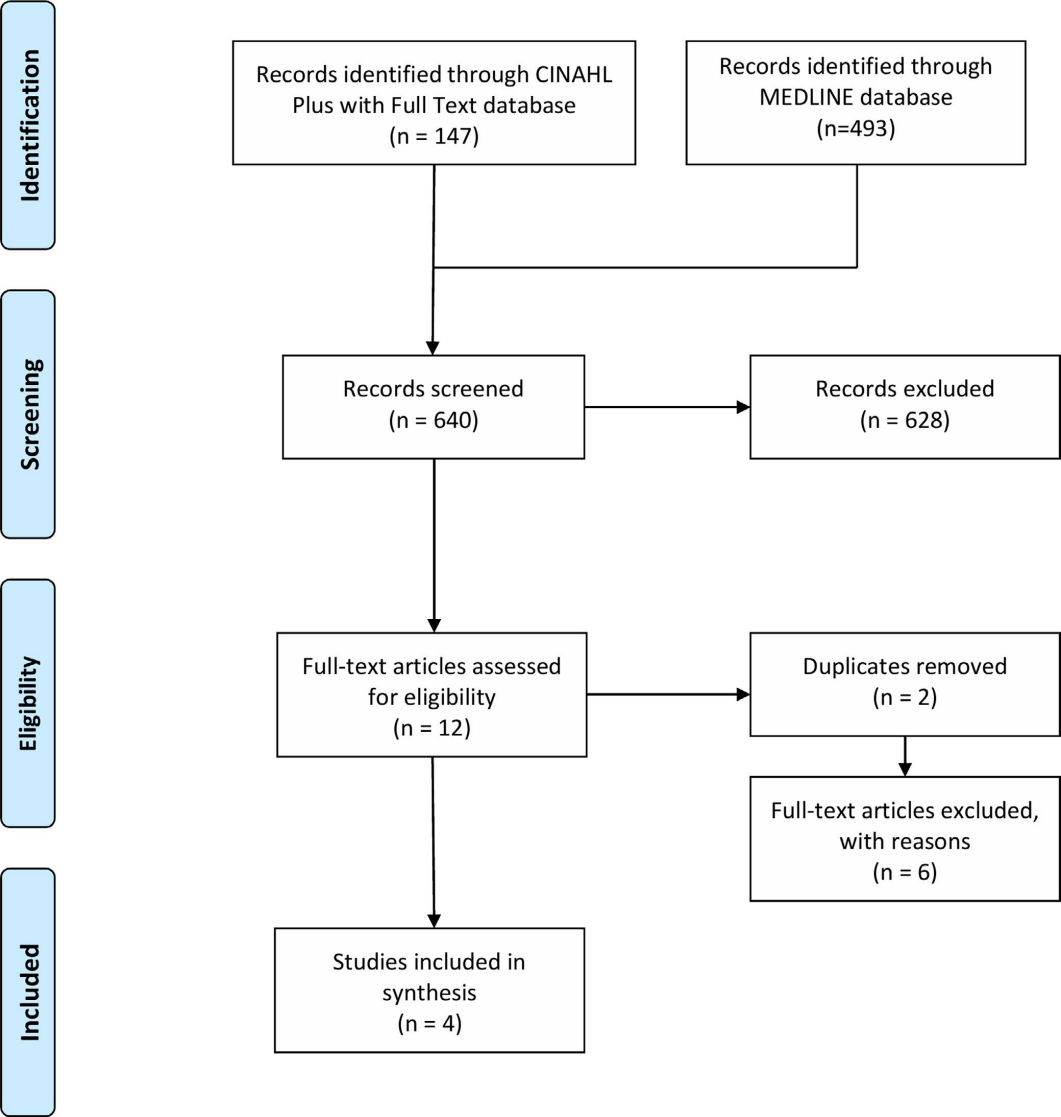
PRISMA flow diagram

There were 147 results identified within the CINAHL database, and 493 results identified within MEDLINE. Six hundred and eight articles were excluded based on title and abstract review. The remaining 12 articles were assessed in full text to determine eligibility based on inclusion and exclusion criteria. Two studies were removed as duplicates, and six studies were excluded based on inclusion/exclusion criteria. These six articles were removed due to being an editorial (Beitz, 2010) or because they did not include discussion of an algorithmic approach (Iqbal et al., 2017; Jon et al., 2008; Lovejoy et al., 1997; Mitchel, 1998; Nagle et al., 2012). After exclusion of ineligible studies, four studies were included in this synthesis. A flow chart of the study selection process can be found in Figure 1.

## 7 QUALITY APPRAISAL

The eligible literature was critically appraised using a tool developed by Hawker et al. (2002), created to analyse both qualitative and quantitative studies. This tool critiques nine items, on a four‐point scale. Each item is rated either good (4), fair (3), poor (2) or very poor (1). Ratings are based on the criteria outlined by Hawker et al. (2002). Summary scores were then calculated overviewing the quality of available evidence.

Studies were critiqued by each author separately to provide researcher triangulation, followed by consensus about ratings through discussion and agreement. The average study score across all literature included within this review was 28.5 out of 36, meaning the average quality of studies was good. Study scores varied from 21 to 35. The highest average rated items were abstract and title, method and data, and implications and usefulness (3.5), while the lowest rated item was ethics and bias (2.25). The remaining average scores for each item critiqued include the following: introduction and aims (3.25), sampling (3), data analysis (3.25), results (3.25) and transferability or generalizability (3). Critical appraisal for each study can be seen in Table 1.

**TABLE 1:** Critical appraisal

Study	1	2	3	4	5	6	7	8	9	Total (36)
	Abstract and title	Introduction and aims	Method and data	Sampling	Data analysis	Ethics and bias	Results	Transferability or generalizability	Implications and usefulness	
Bare et al. (2017)	2	2	3	2	3	1	3	2	3	21
Beitz et al. (2010)	4	4	4	4	3	4	4	4	4	35
Beitz et al. (2014)	4	4	3	3	3	3	4	3	4	31
Kalashnikova et al. (2011)	4	3	4	3	4	1	2	3	3	27
Average Scores	3.5	3.25	3.5	3	3.25	2.25	3.25	3	3.5	28.5

## 8 DATA ABSTRACTION

Data abstraction occurred upon review of all included literature. After reviewing the articles and critiquing their quality, they were placed into a table outlining the authors, country and year the study occurred, the aim of the study, design, methods and instruments used within the study as well as their main findings (Table 2). These data were then used during the synthesis phase of this study utilizing a constant comparison method recommended for integrative reviews by Whittemore and Knafl (2005).

**TABLE 2:** Synthesized studies

Authors, Country and Year	Objective	Design, methods and Instrument	Main findings
Bare, et al United States 2017	To provide a standardized approach to ostomy education and management to support nurses in early identification of stomal and peristomal complications, pouching problems and provide standardized solutions for managing ostomy care in general while improving utilization of formulary products.	Two‐phase quality improvement project using an ostomy algorithm adapted from Beitz et al for the home care setting. Phase 1 was a pilot study of six home healthcare centres in Pennsylvania where the algorithm was used with 20 patients. Phase 2 incorporated the ostomy algorithm into over 300 care centres within the healthcare system.	Although the number of patients increased, costs related to ostomy management were reduced from the year prior. Nursing knowledge, confidence and patient satisfaction were found to have increased in those nurses sampled during project implementation. It was demonstrated that implementation of an ostomy algorithm can be successfully implemented on a large scale.
Beitz, et al United States 2010	To obtain content validation data for the newly developed ostomy algorithm from expert WOCN clinicians.	A cross‐sectional, online, quantitative mixed‐methods survey design with qualitative components was used. A purposive sample of 166 wound, ostomy and continence registered nurses including enterostomal therapy nurses and certified ostomy care nurses was included. The sample included nurses from 40 out of 50 states.	Outcomes support the content validity of the ostomy algorithm developed (overall score was 0.95). The algorithm may assist with optimal care and facilitate standardized ostomy care and documentation. The ostomy algorithm may be beneficial in all clinical settings. Algorithm text was found to be too wordy and patient considerations and preferences were noted to be important. It was felt that many of the eleven assessments overlapped in determining the overall picture of the ostomy patient.
Beitz, Gerlach, & Schafer United States 2014	To evaluate construct validity for a previously face and content validated Ostomy Algorithm using digital real‐life clinical scenarios.	A cross‐sectional, mixed‐methods web‐based survey design was used. A sample of 297 English speaking registered nurses participated in the study.	The mean overall percentage of correct responses using the algorithm was 82.23%. The algorithm may be the first face, content, and construct validated instrument in a digital format. The algorithm was found to be an effective tool for clinicians with a self‐declared basic, intermediate and expert level of ostomy knowledge.
Kalashnikova, et al Russia 2011	To develop a uniform approach to the diagnosis and treatment of ostomy complications that would help optimize rehabilitation of patients with a stoma and be easy for non‐specialty nurses to use.	Prospective evaluation study. Non‐expert nurses utilized the algorithm for 1,427 patients after obtaining education over a two day period.	Ongoing use of the developed algorithm suggests that it assisted with development of a rapid diagnosis and classification of ostomy complications. Stoma complications or peristomal skin disorders were found in 38.8% of the population. The algorithm has not yet been validated.

## 9 SYNTHESIS

A constant comparison analysis method was used in order to present broad categories of credible findings according to Whittemore and Knafl (2005). The five stages to the constant comparison method followed include data reduction, data display, data comparison, conclusion drawing and verification (Whittemore & Knafl, 2005). Data reduction occurred during development of initial study groups including those studies focussed on outlining ostomy management, algorithm implementation and those studies evaluating ostomy algorithms. Data display can be found in Tables 1 and 2 which outline the study information and critique. Data were then compared and final themes developed, followed by a narrative summary of each theme and conclusion.

## 10 RESULTS

The eligible articles were published between 2010 and 2017. The study purposes included content and construct validation of an ostomy algorithm (Beitz et al., 2010, 2014) and development of an ostomy algorithm to assist nurses in identifying and managing ostomy complications (Beitz et al., 2010; Kalashnikova et al., 2011). The participants in the studies included 166 wound, ostomy and continence registered nurses (Beitz et al., 2010), 297 English speaking registered nurses (Beitz et al., 2014), and an undisclosed number of non‐expert nurses who utilized an ostomy algorithm on 1,427 patients (Kalashnikova et al., 2011). In a two‐phase study, the initial phase included 20 patients over six home healthcare settings, while the second phase included 300 care centres (Bare et al., 2017). The sampling techniques used included purposive sampling (Beitz et al., 2010) and representative quota sampling (Beitz et al., 2014). Two studies did not note a sampling method (Bare et al., 2017; Kalashnikova et al., 2011).

The studies occurred in Russia (Kalashnikova et al., 2011) and three within the United States (Bare et al., 2017; Beitz et al., 2010, 2014). Research methodologies included cross‐sectional, mixed‐method survey designs within two studies (Beitz et al., 2010, 2014). One study utilized a prospective evaluation design (Kalashnikova et al., 2011), and one study was a two‐phase quality improvement project (Bare et al., 2017). The data collection methods included surveys (Beitz et al., 2010, 2014), microsoft access/excel (Bare et al., 2017; Kalashnikova et al., 2011) and Cognos Analytic software (Bare et al., 2017).

Two categories were revealed during data analysis and have been categorized according to the following: validation of available ostomy algorithms and implementation of ostomy algorithms in practice. Four themes were then identified within these categories including algorithm validation, identifying underlying causes, focus on accessories and large‐scale implementation.

### 10.1 *Validation* *of ostomy algorithms*

#### 10.1.1 Algorithm validation

Two of the four studies synthesized within this review related to ostomy algorithm validation (Beitz et al., 2010, 2014). Only one validated ostomy algorithm was found during this literature search. The validated algorithm was developed by Beitz and colleagues which has published face, content and construct validation (Beitz et al., 2010, 2014). This algorithm first validated for content by 166 wound, ostomy and continence nurse clinicians self‐identified as ostomy care experts include eleven assessment points (Beitz et al., 2010). The content validity index of the algorithm was 0.95 out of 1 (Beitz et al., 2010). Unfortunately, the full algorithm, including recommendations for management based on assessment outcomes, has not been published, outside of an adapted version by Bare et al. (2017). It is unclear whether the recommendations for management are similar between the algorithm developed by Beitz et al. (2010) and that presented by Bare et al. (2017), as the original management recommendations were never published.

### 10.2 *Implementation* *of ostomy algorithms in practice*

#### 10.2.1 Identifying underlying causes

The algorithm developed by Kalashnikova. et al. (2011) focussed on identification of underlying stoma and peristomal complications and management techniques. Recommendations for pouching systems and accessory use were not specifically provided by the algorithm (Kalashnikova et al., 2011). Treatment methods instead focussed on determining whether surgical management, as opposed to conservative management, should be considered, and whether a change in the ostomy appliance system was required. Recommendations for the pouching system were not provided by the algorithm (Kalashnikova et al., 2011). The algorithm first developed by Beitz et al. (2010) unfortunately does not publish the possible outcomes following the phases of assessment. The Beitz et al. (2010) algorithm does however also focus on identifying potential ostomy complications. Unfortunately, it is unclear whether recommendations are made relating to these complications as to medical and surgical approaches to management, as compared to only making recommendations for pouching systems, as appears to be the case in Bare et al. (2017) in their adaptation of the Beitz et al. (2010) algorithm.

#### 10.2.2 Focus on accessories

Although three of the four studies included in this synthesis focus on the same or an adapted version of the algorithm described in Beitz et al. (2010), only Bare et al. (2017) provided the algorithm in full, showing the outcomes of the algorithm. The algorithm described in Beitz et al. (2010) and Beitz et al. (2014) has not been provided in full, although in the Beitz et al. (2010) article, assessment headings and included tools were provided. In the algorithm adapted by Bare et al. (2017) for the home care setting, it is clear there is a substantial focus on the use of ostomy accessories such as paste and barrier rings. Even in a healthy protruding stoma with no peristomal complications, it has been recommended to use either a barrier ring or paste (Bare et al., 2017). It also appears that the algorithm presented by Bare et al. (2017) only considered two potential ostomy pouching systems, both mouldable systems. Given that the potential outcomes are not presented in the Beitz et al. (2010) and Beitz et al. (2014) articles, it is unclear whether these algorithm approaches to outcomes may change depending on the availability of ostomy pouching products and accessories.

#### 10.2.3 Large‐scale implementation

Bare et al. (2017) identified that they were able to effectively implement an ostomy algorithm tool throughout a system of over 300 home health care centres. Kalashnikova et al. (2011) also described large‐scale use of an ostomy algorithm tool, although, only in one centre. The tool was utilized with 1,427 patients. Issues noted during widespread implementation across multiple sites included the inability to provide onsite education and collect data from patients and staff across the multiple facilities (Bare et al., 2017). It was found that, through the use of an algorithmic approach, providers were able to deliver standardized approaches to care (Bare et al., 2017; Kalashnikova et al., 2011). Kalashnikova et al. (2011) also identified that the use of the algorithmic approach expedited the identification of stoma and peristomal complications. Algorithms implemented across a home health system allowed for high‐quality ostomy care which led to improved patient satisfaction and nursing confidence related to ostomy care (Bare et al., 2017).

The format of these algorithms is also important to discuss. Bare et al. (2017) and Kalashnikova et al. (2011) both discussed implementation of their algorithms with larger populations and both articles presented their algorithmic approaches in visual format. The algorithm discussed in Beitz et al. (2010) had been discussed as potentially confusing for users requiring training. The Beitz et al. (2010) algorithm was developed into a digital algorithm. Unfortunately, the use of a digital format itself was not assessed, nor was how it compares to a visual, poster/paper representation analysed (Beitz et al., 2014).

## 11 DISCUSSION

The majority of ostomy management is provided by non‐specialized nurses (Beitz et al., 2010). It is therefore important to understand the process by which evidence‐based outcomes are derived from clinician assessment to ensure consistent, effective patient care is provided related to their ostomy. With one of the most important outcomes of an ostomy assessment being a safe and effective mechanism to collect effluent, developing an algorithmic process to guide the non‐specialist nurse may assist with improved standardization of care.

In an editorial, Turnbull (2005) outlined a set of key decisions the clinician must make when determining which pouching system to utilize. Turnbull (2005) broke down the pouching system decision into two parts, each with three decisions, which were termed DFS, or Decision For System. The two parts included decisions about the skin barrier, and decisions about the pouch (Turnbull, 2005). The three decisions about the skin barrier include the following: (1) dimension, whether it should be flat or convex, (2) formulation, meaning if it should be standard or extended‐wear, and (3) stoma aperture, if it should be pre‐cut, cut‐to‐fit, or mouldable (Turnbull, 2005). The three decisions about the pouch include the following: (1) the design, whether it is one or two pieces, (2) fabric, and if it is opaque or transparent, and (3) style, if it is a urinary pouch, open‐, or close‐ended pouching system (Turnbull, 2005).

While many of these aspects to determining an appropriate pouching system remain relevant today, it is important to note that recognizing these decisions does not assist the clinician with making the decision. Interestingly, the algorithms found within this review which were designed to assist the clinician with making informed decisions about ostomy management, often did not include many of these decisions. For example, although neither the Beitz et al. (2010) algorithm nor the Kalashnikova et al. (2011) algorithm published recommended pouching outcomes based on the assessment process, Bare et al. (2017) did publish their recommendations. Based on the validated algorithm by Beitz et al. (2010), Bare et al. (2017) made recommendations for a pouching system and accessories based on clinician assessment. This algorithm did not, however, include decision points such as pouch opacity, if convexity is required, or the type of flange such as cut‐to‐fit or pre‐cut (Bare et al., 2017). This may be related to product availability in the trial facilities; however, it limits transferability of the algorithm to other locations with greater pouching options.

The initial phase of the algorithm quality assurance study by Bare et al. (2017) found that an algorithmic approach, in collaboration with an educational session on using the tool, increased nursing confidence and comfort in managing ostomies. Increased confidence and comfort could be of significant benefit for the nursing team. In a 2013 systematic review, it was found that when looking at the education needs of nurses caring for individuals with ostomies, only two studies had been located, one of which found that only 30% of nurses felt they receive enough education to maintain their ostomy skills and knowledge (Recalla et al., 2013). An algorithmic approach may assist with maintaining a standardized approach based on content that is readily accessible when required.

Not captured within this review were several industry‐specific ostomy management algorithms. This was often either due to a lack of publications related to the algorithmic tools, or a focus on the ostomy assessment rather than the algorithmic approach of the tool. The Fit Indicator Tool (Hollister, 2021), Canadian Ostomy Assessment Guide (Convatec, 1998) and Body Check Tool (Coloplast, 2021) will be compared and contrasted along with the algorithmic approaches found during this literature review.

The Fit Indicator Tool (FIT) algorithm developed by Hollister provides a nine‐point assessment tool (stoma type, stoma construction, output, stoma opening, stoma protrusion, peristalsis, peristomal plane, peristomal skin and abdominal tone) that produces a “score” (green—convexity indicated, yellow—convexity should be considered, red—convexity not required and blue—convexity may be used with caution). The Fit Indicator Tool is designed to assist clinicians in determining whether the use of convexity is indicated. The tool is presented in a visual format including a webpage adaptation and a paper enabled version as demonstrated in a poster presentation based on the same nine assessment parameters (Drolshagen et al., 2014). The Fit Indicator Tool like Beitz et al. (2010) and Beitz et al. (2014) provided a methodology for validation. Unlike the Beitz et al. (2010) and Beitz et al. (2014) articles, which focussed on validation, Drolshagen et al. (2014) focussed on inter‐rater reliability, with neither tool testing both validity and reliability. The Fit Indicator Tool was trialled on a total of 98 patients from seven different countries to determine inter‐rater reliability and received an overall Kappa score of 0.63 for the Convexity Guide using a mix of non‐specialized and specialized ostomy nurses indicating substantial agreement (Drolshagen et al., 2014). The algorithm does not provide details of how each assessment criterion was determined; however, the tool website does indicate that the algorithm is based on clinical consensus and developed by a panel of experienced ostomy care nurses from several countries, similar to the development of other industry‐based algorithm development approaches (Hollister, 2021).

The Canadian Ostomy Assessment Guide (COAG), produced in 1998, is the oldest algorithm known to the authors and was only available by hardcopy (ConvaTec, 1998). This guide incorporated multiple algorithms to assist the clinician in determining management of the ostomy including separate algorithms for ileostomy and colostomy management, urostomy management and determining the need for convexity (ConvaTec, 1998). The COAG Convexity Algorithm provides a three point algorithm which asks questions related to the abdominal type, peristomal skin surface and level of stoma retraction if applicable. Unlike other algorithms, the COAG Convexity Algorithm when used in combination with the other COAG algorithms goes beyond just convexity as it is designed to assist clinicians in determining whether accessories are required and whether the pouching system should be a one‐piece or two‐piece pouching system by using icons. The COAG algorithms for urinary pouching systems and faecal pouching systems provide more pouch‐specific recommendations. These algorithms consider twelve assessment points including the following: stoma profile, abdominal contour, stoma shape, skin surface, peristomal skin, devices, limitations, output, quality of life, nursing actions, expected outcome, and provide recommendations if no specific pouching system options are needed. The COAG algorithms contain more assessment points than any other ostomy management algorithm. The assessment points for both the general COAG ostomy assessments and those presented in Beitz et al. (2010) are quite similar as it was noted that the Beitz et al. (2010) algorithm was based on the COAG algorithms and updated literature. Both algorithms consider stoma profile, abdominal contour, stoma shape, skin surface, peristomal skin, devices and output. The Beitz et al. (2010) algorithm does consider the type of ostomy, which is also done in the COAG by separating the algorithms completely based on ostomy type. Beitz et al. (2010) also considered the stoma type, and stoma complications as well as including a peristomal skin tool to determine complication location, while the COAG includes patient limitations, quality of life, nursing actions and expected outcomes (ConvaTec, 1998).

Unlike other algorithms found during this review, the COAG algorithms provide guidance for special circumstances such as devices, pain, dexterity, visual acuity, mobility and cognitive status and output (ConvaTec, 1998). There is another separate algorithm that provides guidance for clinicians on the assessment and treatment of common peristomal skin problems that can be used in tandem with the convexity algorithm. St‐Cyr (2002) evaluated the COAG by recruiting and training 18 community prepared, non‐specialized nurses to utilize the algorithm on 50 patients. Like Bare et al. (2017), St‐Cyr (2002) sought to evaluate pouch longevity and product utilization. The evaluation found that compliance for the COAG went from 56% pre study to 73% during the study, resulting in pouching system wear times increasing from a mean of 4.2 days at baseline to 5.7 days at final visit, a difference of 1.5 days (*p* <.001). This decrease in pouching system use was similar to the findings of Bare et al. (2017), who found that despite an increase in the number of patients seen by 20%, through the use of an algorithmic approach, they decreased the use of skin barriers and pouches by 13.5% year over year. St‐Cyr (2002) also found that the cost per day of ostomy products at baseline was $4.70 and $2.90 at follow‐up, a difference of $1.80 (*p* < .05).

The final industry‐focussed algorithmic tool compared with the findings of the literature search is that of the Body Check Tool developed by Coloplast, which delivers a web‐based eight‐point assessment tool which includes the following: abdominal protrusion, abdominal contour, abdominal firmness, peristomal creasing, stoma location, stoma protrusion, stoma output and pouching preferences (Coloplast, 2021). As with the COAG guide, this tool has similar assessment points to the Beitz et al. (2010) article which also assessed the abdomen, peristomal skin, stoma and output. As with the Beitz et al. (2010) article, it does not include assessment of topics such as quality of life, and patient limitations. The web‐based tool does not provide any insight into how the tool was developed; however, Colwell et al. (2019) published an article funded by Coloplast which outlines similar assessment parameters as the Body Check Tool. Colwell et al. (2019) included shape and diameter as an added assessment parameter, did not include pouching preference, and expanded on lumen opening and protrusion as different assessment parameters instead of one assessment parameter which was combined in the Body Check Tool (Coloplast, 2021). Colwell et al. (2019), in development of assessment points, performed a three‐stage modified Delphi consensus‐building process using an international group of non‐specialized and specialized ostomy nurses and an expert review group of 15 specialized nurses including two Canadian nurses. Unlike Bare et al. (2017), Kalashnikova et al. (2011), Beitz et al. (2010) and Beitz et al. (2014), which are intended to be used by non‐specialized and specialized ostomy clinicians, the Body Check algorithm, based on the Colwell et al. (2019) assessment criteria, has the intended audience for the Body Check Tool as the end user and its purpose is to engage and educate patients.

## 12 LIMITATIONS

The search strategy utilized within this review may be considered the main limitation of the manuscript. Standardized guidelines for conducting systematic reviews, such as the PRISMA Statement for reporting systematic reviews, recommend a minimum of at least one database be searched during the review process (Liberati et al., 2009). Whittemore and Knafl (2005), however, note that they believed approximately only 50% of studies may be found within research databases. It should also be noted that studies were limited to English, given that the researchers were only able to review and critique in English; therefore, there may be non‐English studies which have not been included within this review. To increase the rigour in the literature search, no timelines were included in the search, broad terms for algorithms were utilized, and outcomes have been presented in a structured format which can be reproduced. Rigour was further improved using techniques outlined by Whittemore and Knafl (2005) including the following: use of the constant comparison data analysis method, providing a clear inclusion and exclusion criteria and clearly outlining database search methods.

It should also be noted that two studies validating the same algorithm were industry funded by an ostomy company who provides to the study authors employment, speaker honoraria, research grant funding and/or are consultants or paid advisory board members (Beitz et al., 2010, 2014). The initial algorithm validation study was funded by the ostomy company and provided editorial support for the publication (Beitz et al., 2010). This was the only validated algorithm found within study findings. The Bare et al. (2017) study included within the findings utilized a modified version of the algorithm validated with industry funding.

Future research is required to understand the evidence‐based ostomy assessment process and decision‐making procedure for healthcare professionals managing an ostomy. New algorithms should be developed encompassing a broad range of outcome recommendations related to pouching system management. Further testing of ostomy management algorithms is required using high‐level research designs to confirm the potential benefits of use.

## 13 CONCLUSION

Current literature has demonstrated a paucity of available validated ostomy management algorithms. No validated, fully published algorithms for ostomy management were found during the literature search for this review. Only one algorithm, which both provided direction on assessment and included management outcomes, was present, and the outcomes were heavily focussed on accessory use, with only mouldable appliances included in the outcomes.

The current literature available providing algorithmic approaches to ostomy management has often been created using manufacturer‐specific products which have since either become obsolete or will in the future as technology advances. While the use of convexity is recommended, all available algorithms stop short in providing more specific recommendations such as the type or depth of convexity required (i.e. soft, light and deep). This lack of specificity places the pouching system decision into the hands of the non‐specialized clinician without clear guidance.

Current evidence has demonstrated the potential for ostomy management algorithms to standardize care across multiple organizations and facilities with large numbers of patients. Algorithms may also improve recognition of ostomy complications, increase patient satisfaction and improve nurse confidence. While further research with high‐level research designs is required to demonstrate the effects of ostomy management algorithms, current evidence provides a foundation for the possibilities of use in clinical practice.

## CONFLICT OF INTEREST

No conflicts of interest to declare.

## AUTHOR CONTRIBUTIONS

All authors have agreed upon the final version of the manuscript. All authors have provided substantial contributions to the conception and design or analysis and interpretation of the data. All authors have participated in drafting or critically revising the manuscript for important intellectual content. All authors agree to be accountable for all aspects of the work.

## Data Availability

The authors confirm that the data supporting the findings of this study are available within the article.
